# Heterogeneity in the costs of type 1 diabetes in a developing country: what are the determining factors?

**DOI:** 10.1186/1758-5996-5-83

**Published:** 2013-12-27

**Authors:** Roberta Arnoldi Cobas, Marcos Bosi Ferraz, Alessandra Saldanha de Mattos Matheus, Lucianne Righeti Monteiro Tannus, Aline Tiemi Kano Silva, Luiz Antonio de Araujo, Carlos Antonio Negrato, Sérgio Atala Dib, Marilia Brito Gomes

**Affiliations:** 1 Disciplina de Diabetes, State University Hospital of Rio de Janeiro (UERJ), Rio de Janeiro, Brazil- Avenida 28 de setembro, 77, Terceiro andar, Vila Isabel 20551-030, Brazil; 2 Federal University of São Paulo (UNIFESP), São Paulo, Brazil- Rua Botucatu, 685, Vila Mariana, Brazil; 3 Bauru’s Diabetics Association, Brazil- Avenida Nações Unidas, 28-40, Bauru 17011-105, Brazil; 4 Joinville Endocrinology and Diabetes Institute, Santa Catarina, Brazil- Rua Alexandre Dohler 129, Joinville 89201-260, Brazil

**Keywords:** Type 1 diabetes, Direct costs of type 1 diabetes, Chronic complications

## Abstract

**Background and aims:**

Regional differences in the clinical care of Type 1 diabetes (T1D) in Brazil have been recently described. This study aimed to estimate the costs of T1D from the public health care system’s perspective across the regions of Brazil and to determine the components that influence these costs.

**Methods:**

This was a retrospective, cross-sectional and nationwide multicenter study conducted between December 2008 and December 2010 in 28 public clinics in 20 Brazilian cities. The study included 3,180 T1D subjects receiving healthcare from the National Brazilian Healthcare System (NBHCS) with a follow-up of at least one year. The direct medical costs were derived from the costs of medications, supplies, examinations, visits to the center, medical procedures and hospitalizations that occurred during the previous year. Clinical and demographic factors that determined the differences in the cost across four geographic regions (southeast, south, north/northeast and mid-west) were investigated.

**Results:**

The *per capita* mean annual direct medical costs of T1D in US$ were 1,466.36, 1,252.83, 1,148.09 and 1,396.30 in southeast, south, north/northeast and mid-west regions, respectively. The costs of T1D in the southeast region were higher compared to south (p < 0.001) and north/northeast regions (p = < 0.001), but not to the mid-west (p = 0.146) region. The frequency of self-monitoring of blood glucose (SMBG) was different across the regions as well as the daily number of SMBG, use of insulin pumps or basal or prandial insulin analogs. Age, ethnicity, duration of diabetes, level of care, socioeconomic status and the prevalence of chronic diabetic complications differed among the regions. In a regression model the determinants of the costs were the presence of microvascular diabetes-related complications (p < 0.001), higher economic status (p < 0.001), and being from the southeast region (p < 0.001).

**Conclusions:**

The present data reinforce the regional differences in the costs of T1D and in the socioeconomic profile and health care provided to the patients with T1D in specialized public centers in Brazil. Both factors influenced directly the costs of T1D and should be considered for discussing future health policies.

## Background

Type 1 diabetes (T1D) is a chronic disease that carries a large risk of chronic disabling complications that have a negative impact in the costs of the disease and in the patient’s quality of life. Moreover, the incidence of T1D is increasing in many countries, including Brazil [1,2]. Despite the well known benefits of an intensive glucose control in reducing or postponing the risks of diabetes-related complications and the costs of the disease, the glycemic control in the majority of patients with T1D in Brazil does not meet the guidelines recommendations [3].

Brazil is a large country with about 191 · 8 million inhabitants, according to the last population census conducted by the Brazilian Institute of Geography and Statistics (IBGE) [4]. It is divided into five major geographic regions (north, northeast, mid-west, southeast, and south) and the proportion of people living in urban areas is of 84% [4]. The regional population densities present wide disparities. The north region comprises 45 · 2% of the total area of the country and has only 8 · 1% of the total population. In contrast, the southeast region accounts for 42% of the total population density and comprises only 10 · 9% of the total area of the country [4]. In addition to demographic differences, cultural, and socioeconomic aspects also differ among regions. Actually, the functional illiteracy rate in people older than 14 years was 23 · 1% in the north, 30 · 8% in the northeast, 15 · 2% in the southeast, 15 · 5% in the south, and 18 · 5% in the mid-west in 2010 [5].

The direct costs of T1D has been recently estimated in Brazil [6]. Previous data from our group have shown important regional differences in the clinical care of T1D patients in Brazil regarding the achievement of therapeutic goals, frequency of screening for chronic diabetes-related complications, insulin regimens, and frequency of self-monitoring of blood glucose (SMBG) [7]. The results found in this study are alarming and suggest that governmental health policy should be directed to each geographic region, in order to meet their specific demands and improve the quality of care in the public health care system overall.

The aim of our study was to estimate the costs of T1D from the public health care system’s perspective across the different regions of Brazil and to determine the regional differences of the components that influence these costs. We believe these data may provide important clues for reassessment of regional health policies in Brazil.

## Research design and methods

### Study design

**Figure 1:**
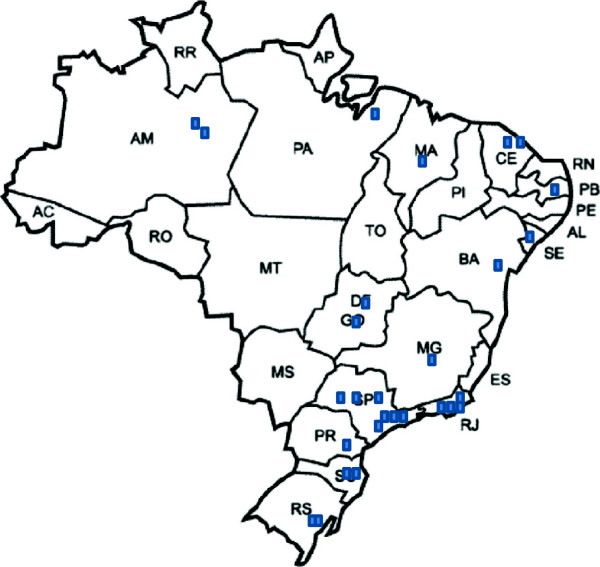
Distribution of the participating centers across the country.

This is a retrospective, cross-sectional and nationwide multicenter cost-of-illness study conducted between December 2008 and December 2010 in 28 centers located in 20 Brazilian cities in public clinics with secondary and tertiary care levels (Figure 1). The public healthcare system in Brazil is divided in primary, secondary and tertiary care levels according to the characteristics and infrastructure of the healthcare units. As patients with T1D usually are treated at secondary or tertiary centers in our country, primary care centers were not included in the study. Also, patients attending the private or supplementary health care system (about 24% of the total Brazilian population) were not included. The detailed methodology has been described elsewhere [3]. Written informed consent for the study was obtained from all of the patients aged 18 years or older or from the parents or guardians of the patients younger than 18 years. The study was approved by each local center’s ethics committee. Only the patients with at least 12 months of follow-up at the respective center were included. This inclusion criterion allowed us to quantify the variables required to determine the costs over the prior year to the study through an interview.

Economic status was defined according to the Brazilian Economic Classification Criteria (ABEP) [8], which estimates the purchasing power of urban individuals and families, thus classifying the population in terms of economic strata. These criteria provide scores according to the ownership of items and education level. Economic status were classified as high, middle, low or very low [8].

### Assessment of clinical and demographic variables

Information on the diabetic treatment modalities, source of insulin pumps, medications and supplies for SMBG, frequency of SMBG, routine diabetes care and other data not available from medical records (eg. admissions in other hospitals) were obtained through an interview with information provided directly by the patient or his caregiver. The following information was obtained from medical records assessed during the interview: the total number of HbA1c measurements performed in the prior year, fructosamine levels, fasting, and 2-h postprandial glycemia, total cholesterol, LDL-cholesterol, HDL-cholesterol, triglycerides, uric acid, plasma creatinine, plasma urea, sodium, potassium, liver enzymes (aspartate [AST] and alanine [ALT] aminotransferase), C-reactive protein (CRP), thyroid stimulating hormone (TSH), proteinuria, and albuminuria. The number of tests performed to screen for diabetes-related complications, such as electrocardiograms (ECGs), exercise stress tests, stress echocardiographs, calcium score tomographies, coronary artery angiographs, and fundoscopies, were analyzed. The frequencies of medical procedures, such as vitrectomy, laser therapy, hemodialysis, and hospitalizations due to diabetes decompensation or ketoacidosis were also evaluated.

### Costs evaluation and distribution of healthcare resources

The direct medical costs were calculated using the costs of medications (oral drugs and insulin), SMBG supplies, blood analysis and other examinations, consultations with physicians, nurses and dietitians, medical procedures, and hospitalizations that occurred during the prior year. The drugs included in the analysis were those specific to treating T1D or its related comorbidities (dyslipidemia, arterial hypertension, obesity), and complications (retinopathy, nephropathy, and neuropathy). The medication costs were obtained from the Brazilian Ministry of Health website [9] when the drug was provided by public institutions or by the average price at three nationwide pharmaceutical web sites when privately acquired. When the price varied by region, we calculated the average cost. The costs of examinations and medical procedures were obtained from the table of procedures using the 2010–2011 NBHCS prices which determine the reimbursements to government-sponsored healthcare units. The price of insulin pumps and their supplies was based on the value paid by the Municipal Health Bureau of Rio de Janeiro (RJ-SMS) in May 2010. The costs of SMBG supplies, syringes, and needles were determined by the April 2011 RJ-SMS or by the mean values for three nationwide pharmaceutical companies when privately acquired.

All of the costs were converted into US dollars (at 1 US dollar = 1 · 9315 Reais) using the average of the 3 consecutive years (2008–2010).

### Data storage and statistical analysis

The statistical analysis was performed using the *Statistical Package for the Social Sciences* (SPSS) Version 17.0.

Data are presented as mean [95% confidence interval] for continuous variables and as counts (relative frequencies) for discrete variables.

Comparison of the direct medical costs across the four regions was performed using non- parametric test Mann–Whitney for every two regions. The statistically significant p-values were corrected using the Sidak procedure to control for type I errors (p < 0 · 01). Data on the north and northeast regions were evaluated together, so four geographic regions were compared (southeast, n = 1,310; south, n = 727; north/northeast, n = 938, and mid-west, n = 205). A regression analysis was performed to investigate the role of the geographic regions and other independent variables (age, ethnicity, duration of diabetes, economic status, level of attention, and the presence of microvascular or macrovascular diabetes-related complications) in influencing the direct medical costs. In this regression analysis the geographic regions were introduced in the model as dummies variables.

## Results

The clinical and demographic characteristics of the studied populations are presented in Table 1. The *per capita* mean [95% IC] direct medical costs of T1D were US$ 1,466 · 36 [1,382 · 09-1,550 · 64], US$ 1,252 · 83 [1,168 · 66-1,337 · 00], US$ 1,148 · 09 [1,048 · 86-1,247 · 32], and US$ 1,396 · 30 [1,268 · 30-1,370 · 00] in southeast, south, north/northeast, and midwest regions, respectively. The costs of T1D in the southeast region were higher compared to the south (p < 0 · 001), and north/northeast regions (p < 0 · 001), but not to the mid-west region (p = 0 · 146). Midwest costs were statistically different from north/northeast (p < 0 · 001) but not from south regions (p = 0 · 292). South costs were higher than north/northeast (p < 0 · 001) (Table 2).

**Table 1:** Clinical and demographic data of the studied population

**Variable**	
**N**	3,180
**Female gender, n (%)**	1,791 (56 · 3)
**Age, y**	22 ± 11 · 8; 20 (2–66)
**Ethnicity, n (%)**	
Caucasian	1,824 (57 · 4)
Non-Caucasian*	1,356 (42 · 6)
Socioeconomic status**	
High	222 (7 · 2)
Medium	710 (22 · 3)
Low	1,052 (33 · 1)
Very low	1,102 (34 · 7)
**Level of care, n (%)**	
Secondary	897 (28 · 2)
Tertiary	2,283 (71 · 8)
**Duration of diabetes, y**	10 · 3 ± 8 · 03, 8 (1–50)
**HbA1c%, mmol/mol**	9 · 34 ± 2 · 34, 78.6 ± 25
**Presence of microvascular complications**^**†**^	635 (27 · 4)
**Presence of macrovascular complications**^**†**^	119 (5 · 1)

y = year. The data are presented as number (percentage), mean ± SD and/or median (Min-Max). *Afro-Brazilians, Mulattos, Asians, Native Indians **Missing data from 87 participants.

^†^Excludes the patients without criteria for the diabetic complications screening.

**Table 2:** Total annual costs (US$) of the studied population across the different geographic regions in Brazil

	***Southeast (n = 1310)***	***South (n = 727)***	***North/Northeast (n = 938)***	***Mid-West (n = 205)***
**Direct medical cost (US$)**				
**Total**	1,920,936 · 74	910,808 · 38	1,076,906 · 08	286,241 · 19
***Per capita***	1,466 · 36	1,252 · 83	1,148 · 09	1,396 · 30
	[1,382 · 09–1,550 · 64]	[1,168 · 66–1,337 · 00]	[1,048 · 86–1,247 · 32]	[1,268 · 30–1,370 · 00]
	1,052 · 10	885 · 54	654 · 86	986 · 42
	(778 · 65–1,710 · 46)	(698 · 01–1,513 · 22)	(429 · 77–1,164 · 17)	(631 · 81–1,871 · 78)

Data presented as mean [95% CI] and median (interquartile range). Southeast vs south (p < 0 · 001); southeast vs north/northeast (p < 0 · 001); southeast vs midwest (p = 0 · 146); midwest vs north/northeast (p = <0 · 001); south vs northeast (p < 0 · 001); south vs midwest (p = 0 · 292).

The components of the clinical care of diabetes that most impacted the direct medical costs were compared among the regions aiming to find explanations for the regional differences of these costs (Table 3). As previously shown, the frequency of SMBG is different across the regions (p < 0 · 001) as well as the daily number of SMBG (p < 0 · 001), use of insulin pumps (p = 0 · 009) or basal or prandial insulin analogs (p < 0 · 001).

**Table 3:** Differences in the resources utilization among the geographic regions of Brazil

	***Southeast***	***South***	***North/Northeast***	***Mid-West***	***p-value***
**SMBG (yes), (%)**	93 · 9	92 · 7	77 · 2	95 · 1	<0 · 001
Daily frequency of SMBG*	3 · 88 [3 · 78–3 · 98] 4 (3–5)	3 · 02 [2 · 91–3 · 13] 3 (2–4)	2 · 93 [2 · 76–3 · 10] 2 (2–3)	3 · 19 [3 · 00–3 · 37] 3 (2–4)	<0 · 001
**Use of insulin pump, n (%)**	23 (1 · 8)	10 (1 · 4)	2 (0 · 2)	3 (1 · 5)	0 · 009
**Use of ultra-rapid insulin analogs, n (%)**	627 (52 · 5)	364 (52 · 4)	122 (16 · 8)	98 (52 · 7)	<0 · 001
**Use of basal insulin analogs n (%)**	281 (21 · 5)	156 (21 · 5)	108 (11 · 5)	73 (35 · 6)	<0 · 001

SMBG = self- monitoring of blood glucose. *Data presented as mean [95%CI] and median (interquartile interval).

The clinical and demographic variables that could have influenced the costs were compared among the regions and the results are shown in Table 4. Age (p = 0 · 017), ethnicity (p < 0 · 001), duration of diabetes (p < 0 · 001), level of care (p < 0 · 001), socioeconomic status (p < 0 · 001) and the prevalence of chronic diabetes-related microvascular (p < 0 · 001) and macrovascular (p = 0 · 004) complications differed among the regions but not gender.

**Table 4:** Clinical and demographic differences among the geographic regions of Brazil

	***Southeast***	***South***	***North/Northeast***	***Midwest***	***P value***
**Gender (female), (%)**	58 · 5	56 · 3	53 · 1	57 · 6	0 · 087
**Ethnicity (Caucasian), (%)**	58 · 7	87 · 3	34 · 6	46 · 3	<0 · 001
Age (y)*	22 · 0 ± 12 · 5	23 · 4 ± 12 · 3	20 · 9 ± 9 · 9	21 · 8 ± 11 · 9	0 · 017
Duration of diabetes (y)**	11 · 2 ± 8 · 8	11 · 4 ± 8 · 3	8 · 5 ± 6 · 4	9 · 4 ± 7 · 5	<0 · 001
Level of care*** (tertiary), (%)	93 · 1	94 · 2	34 · 9	24 · 9	<0 · 001
Low or very-low economic status (%)****	66 · 1	58 · 1	86 · 1	58 · 2	<0 · 001
**Prevalence of microvascular complications (%)**	22 · 0	23 · 8	14 · 5	18 · 5	0 · 001
**Presence of macrovascular complications (%)**	5 · 1	3 · 2	2 · 5	2 · 9	0 · 004

SE = Southeast; N/NE = North/Northeast; MW = Midwest.

*p = 0 · 006 (SE vs South), p = 0 · 003 (South vs N/NE).

**p <0 · 001 (SE vs N/NE), p = 0 · 006 (SE vs MW), p <0 · 001 (South vs N/NE), p = 0 · 001 (South vs MW).

***p <0 · 001 (SE vs N/NE), p <0 · 001 (SE vs MW), p <0 · 001 (South vs N/NE), p <0 · 001 (South vs MW), p = 0 · 007 (N/NE vs MW).

****p <0 · 001 (N/NE vs MW), p = 0 · 042 (SE vs MW),p <0 · 001 (SE vs N/NE), p <0 · 001 (SE vs South), p = 0 · 02 (South vs MW), p <0 · 001 (N/NE vs South).

In the regression model using the direct medical costs as the dependent variable and geographic region, age, ethnicity, duration of diabetes, level of attention, socioeconomic status and the presence of chronic microvascular or macrovascular diabetes-related complications as the independent variables we observed that the independent determinants of the costs were the presence of microvascular diabetes-related complications (B -603 · 00, SE 64 · 36; β -0 · 165; p < 0 · 001), economic status (B -202 · 57, SE 27 · 72; β -0 · 129; p < 0 · 001), and being from the southeast region (B 197 · 95, SE 52 · 77; β 0 · 066; p < 0 · 001). This model explained only 5 · 4% of the variability of the costs (R^2^ = 0 · 054).

## Discussion

Brazil is a large country with a great cultural, socioeconomic and demographic diversity. As previously demonstrated [7], these diversities are also extrapolated to the type of treatment and clinical care of T1D patients in our country. From the present data we can see that the direct medical costs of T1D also follow this same pattern.

The first interesting issue raised by the present study is the existence of two extremes in Brazil regarding the characteristics, the care of patients with T1D and the costs of the disease: the southeast and the north/northeast regions. In the southeast region, the average *per capita* costs of T1D was 28% and 17% higher than in the north/northeast and in the south regions, respectively, although not different from mid-west. In fact, being from the Southeast region was independently associated with higher medical costs. This finding could be explained by the different pattern of treatment offered to patients living and receiving treatment in each region which is reflected in the costs. For example, a smaller proportion of patients in the north/northeast region performed SMBG and when performed, it was in a lower daily frequency. Moreover, the great majority of patients were using the lower cost human regular or NPH insulins. However, we also observed that most patients in north/northeast belong to low or very low socioeconomic status and are seen mostly in secondary centers.

Another determinant of the costs of T1D in our study was the socioeconomic status. What must be investigated is 'how far it, which also reflects the educational level in the ABEP classification, interferes with the costs’? Intuitively, we can consider that both the patient and the healthcare team can act to determine this difference. In the daily clinical practice physicians know that the complexity of the treatment offered to the patients should correspond to their acceptance and ability to follow the recommendations adequately. Otherwise, it would result in poor compliance and any proposed treatment strategy would be inefficient. This means that we are increasingly individualizing treatment in diabetes and this has already reflected in the most recent guidelines recommendations for type 2 diabetes (T2D) treatment [10]. These recommendations consider the patients’ motivation, adherence, and self-care capacities as well as the resources and support system, as important factors in choosing the best treatment for a particular patient. On the other hand, patients with a higher education or even cognitive level could act more positively in finding the best available treatment options for him, many of which, are more expensive. This is particularly important in many cities in Brazil where the public health care system do not provide insulin analogs and the supplies for SMBG in a regular basis.

Other determinant of higher costs in T1D is the presence of chronic diabetes-related microvascular complications. In epidemiological studies, it is important to consider that the prevalence of chronic complications is proportional to the frequency of screening and the survival to acute complications of the disease. Patients from north/northeast presented lower prevalence of complications, which could reflect a survival bias. Intervention studies, such as the Diabetes Control and Complications Trial (DCCT), have shown that intensive treatment during the early stages of T1D reduces the risk of future microvascular complications [11]. Information derived from clinical and economic trials can guide economic policy decisions that aim to reduce direct costs by reallocating resources toward preventing acute and chronic diabetes-related complications. Increased costs due to the presence of chronic diabetes-related complications may have a future impact on health economics because chronic complications become more prevalent as the disease progresses.

The impact of T1D alone on the total investment in the public health care system by the three spheres of government, Federal, State and Municipal is unknown. According to data from the National Health Ministry of Brazil we have observed an increase in global health expenditure from years 2008 to 2010 in all regions of the country, which is determined by law. The *per capita* expenditure is higher in the southeast and lowest in the north/northeast regions (Table 5), consistent with the trend of the *per capita* costs of T1D. However, patients with T1D cost about 4 times the *per capita* expenditure on public health services and actions by the Ministry of Health in all regions. The fixed offer and the variable demand could explain these similar relations despite the differences in regional costs. Thus, it is likely that the health investment cap would be a limiting factor in the *per capita* costs of T1D.

**Table 5:** ***Per capita *** **expenditure on public health services and actions for each geographic region according to the National Health Ministry of Brazil**

**Per capita expenditure (US$/% Total)**	***2008***	***2009***	***2010***	***3-year mean***	***Individual cost of T1D/Per capita expenditure***
**North/Northeast**	237 · 20 (21 · 8%)	256 · 89 (22 · 0%)	282 · 23 (21 · 7%)	258 · 77 (21 · 8%)	4 · 44
**Southeast**	306 · 17 (28 · 2%)	331 · 73 (28 · 5%)	373 · 87 (28 · 7%)	337 · 26 (28 · 5%)	4 · 35
**South**	257 · 89 (23 · 7%)	279 · 12 (23 · 9%)	321 · 06 (24 · 7%)	286 · 02 (24 · 2%)	4 · 38
**Midwest**	285 · 15 (26 · 3%)	297 · 86 (25 · 6%)	323 · 52 (24 · 9%)	302 · 18 (25 · 5%)	4 · 62

Although the study design does not allow a complete evaluation of the clinical and economic consequences of the treatment of T1D in Brazil, we suspect that other factors besides the economic status may interfere in clinical outcomes and in meeting the recommended therapeutic goals. For instance, the southeast and mid-west regions, despite having a 22% and 28% higher T1D *per capita* costs, respectively, a greater frequency of SMBG and proportion of insulin analogues use than the north/northeast region, presented a similar low prevalence of patients within the recommended HbA1c targets [7].

Some study limitations must be addressed. The study sample included only patients attended at public centers on secondary or tertiary levels in urban areas. However, according to the structure of care established by the NBHCS, the vast majority of patients with T1D are treated in these centers. Also, the collection of data may have led to collection bias.

The present data reinforce the regional differences in the health care provided to patients with T1D in specialized public centers in Brazil, as well as the regional differences in the socioeconomic and demographic profile of the population. Both factors have directly influenced the costs of T1D in Brazilian regions and should be considered for discussing future health policies.

## Competing interests

Authors declare that they have no conflicts of interests.

## Author’s contributions

RAC, ASMM, LRMT researched data and drafted the manuscript. ATKS, LAA, SAD researched data. MBF contributed to conception and design, reviewed the manuscript and contributed to discussion. RAC, CAN and MBG reviewed the manuscript and contributed to the discussion. The writing group takes final responsibility for the paper and is the study guarantor. All authors read and approved the final manuscript.

## Author’s informations

*Brazilian Type 1 Diabetes Study Group (BrazDiab1SG).

Executive steering committee: Marilia Brito Gomes (chair), Roberta Cobas, Sergio Atala Dib, Carlos Negrato.

Principal investigators are indicated by an asterisk. Program coordinators are italic.

Universidade Estado Rio de Janeiro: Roberta Cobas*, *Alessandra Matheus*, Lucianne Tannus; Universidade Federal Rio de Janeiro: Melanie Rodacki*, *Lenita Zadenverg*; Hospital Geral de Bonsucesso: Neuza Braga Campos de Araújo*, *Marilena de Menezes Cordeiro*; Hospital Universitário Clementino Fraga Filho – IPPMG: Dr. Jorge Luiz Luescher*; *Renata Szundy Berardo*; Serviço de Diabetes da Disciplina de Endocrinologia e Metabologia do Hospital das Clínicas da Universidade de São Paulo: Marcia Nery*; *Catarina Cani*; Maria do Carmo Arruda Marques; Unidade de Endocrinologia Pediátrica da Santa Casa de Misericórdia de São Paulo: Luiz Eduardo Calliari*, *Renata Maria de Noronha*; Instituto da Criança do Hospital das Clínicas da Universidade de São Paulo: Thais Della Manna*, *Roberta Salvodelli*, Fernanda Garcia Penha; Hospital das Clínicas da Faculdade de Medicina de Ribeirão Preto – USP: Milton Cesar Foss*, *Maria Cristina Foss-Freitas*; Ambulatório da Faculdade Estadual de Medicina de São José do Rio Preto: Antonio Carlos Pires*, *Fernando Cesar Robles*; Associação de Diabéticos de Bauru: Carlos Antonio Negrato*, *Maria de Fatima Guedes*; Centro de Diabetes da Escola Paulista de Medicina: Sergio Atala Dib*, *Patricia Dualib*; Clínica de Endocrinologia da Santa Casa de Belo Horizonte Setor Diabetes Tipo 1: Saulo Cavalcanti da Silva*, *Janice Sepulveda*; Ambulatório Multiprofissional de Atendimento à Diabetes do Hospital de Clínicas da Universidade Estadual de Londrina: Henriqueta Guidio de Almeida*, *Emerson Sampaio*; Hospital de Clínicas da Universidade Federal do Paraná:Rosangela Roginski Rea*, *Ana Cristina Ravazzani de Almeida Faria*; Instituto da Criança com Diabete Rio Grande Sul: Balduino Tschiedel*, *Suzana Lavigne*, Gustavo Adolfo Cardozo; Hospital de Clínicas de Porto Alegre:Mirela Azevedo*, *Luis Henrique Canani*, Alessandra Teixeira Zucatti; Hospital Universitário de Santa Catarina: Marisa Helena Cesar Coral*, *Daniela Aline Pereira*; Instituto de Diabetes-Endocrinologia de Joinville: Luiz Antonio de Araujo*; Hospital Regional de Taguatinga, Brasília: Hermelinda Cordeiro Pedrosa*, *Monica Tolentino*; Flaviene Alves Prado; Hospital Geral de Goiânia: Dr Alberto Rassi: Nelson Rassi*, *Leticia Bretones de Araujo*; Centro de Diabetes e Endocrinologia do Estado da Bahia: Reine Marie Chaves Fonseca*; *Alexis Dourado Guedes*, Odelisa Silva de Mattos; Universidade Federal do Maranhão: Manuel Faria*, *Rossana Azulay*; Centro Integrado de Diabetes e Hipertensão do Ceará: Adriana Costa e Forti*, *Maria Cristina Façanha*; Universidade Federal do Ceará: Renan Montenegro Junior*, *Ana Paula Montenegro*; Universidade Federal de Sergipe: Naira Horta Melo*, *Karla Freire Rezende*; Hospital Universitário Alcides Carneiro: Alberto Ramos*; Hospital Universitário João de Barros Barreto, Pará: João Felício Soares*, *Flavia Marques Santos*; Hospital Universitário Getúlio Vargas, Hospital Adriano Jorge: Deborah Laredo Jezini*.

## References

[B1] Sr F Franco LJ Vivolo MA Negrato CA Simoes AC Ventureli CR Population-based incidence of IDDM in the State of São Paulo, Brazil Diabetes Care 1993 5 701 04 10.2337/diacare.16.5.701 8495607

[B2] Negrato CA Dias JP Teixeira MF Dias A Salgado MH Lauris JR Montenegro RM Jr Gomes MB Jovanovic L Temporal trends in incidence of type 1 diabetes between 1986 and 2006 in brazil J Endocrinol Invest 2010 5 373 77 19620822 10.1007/BF03346606

[B3] Gomes MB Coral M Cobas RA Dib SA Canani LH Nery M De Freitas MC Faria M Felício JS Da Silva SC Pedrosa H Costae Forti A Rea RR Pires AC Montenegro Junior R Oliveira JE Rassi N Negrato CA Prevalence of adults with type 1 diabetes who meet the goals of care in daily clinical practice: a nationwide multicenter study in brazil Diabetes Res Clin Pract 2012 5 63 70 10.1016/j.diabres.2012.02.008 22397904

[B4] Instituto Brasileiro de Geografia e Estatística Censo demográfico 2010 2010: Available from: http://www.ibge.gov.br/home/estatistica/populacao/censo2010/caracteristicas_da_populacao/tabelas_pdf/tab1.pdf. Accessed 15 March 2012

[B5] Instituto Brasileiro de Geografia e Estatística (2010) Uma análise das condições de vida da população brasileira 2010 [http://www.ibge.gov.br/home/estatistica/populacao/condicaodevida/indicadoresminimos/sinteseindicsociais2010/default.shtm]

[B6] Cobas RA Ferraz MB Matheus ASM Tannus LRM Negrato CA Araujo LA Dib SA Gomes MB The cost of type 1 diabetes: a nationwide multicentre study in Brazil Bull World Health Organ 2013 5 434 40 10.2471/BLT.12.110387 24052680 PMC3777141

[B7] Gomes MB Cobas RA Matheus ASM Tannus LRM Negrato CA Rodacki M Braga N Cordeiro MM Luescher JL Berardo RS Nery M Arruda-Marques MC Calliari LE Noronha RM Manna TD Zajdenverg L Salvodelli R Penha FG Foss MC Foss-Freitas MC Pires AC Robles FC Guedes M Dib SA Dualib P Silva SC Sepulvida J Almeida HG Sampaio E Rea R Regional differences in clinical care among patients with type 1 diabetes in Brazil: Brazilian type 1 diabetes study group Diabetol Metab Syndr 2012 5 44 doi:10.1186/1758-5996-4-44 10.1186/1758-5996-4-44 23107314 PMC3538646

[B8] ABEP Brazilian Economic classification criteria 2012 [http://www.abep.org/novo/Content.aspx?SectionID=84]

[B9] Portal da saúde (SUS) Banco de preços em saúde 2011 [http://portal2.saude.gov.br/BPS/visao/consultapublica/publico_interno_item.cfm]

[B10] Inzucchi SE Bergenstal RM Buse JB Diamant M Ferrannini E Nauck M Peters AL Tsapas A Wender R Matthews DR Management of hyperglycemia in type 2 diabetes: a patient-centered approach. Position statement of the American diabetes association (ADA) and the European association for the study of diabetes (EASD) Diabetes Care 2012 5 1364 1379 10.2337/dc12-0413 22517736 PMC3357214

[B11] DCCT The diabetes control and complications trial N Engl J Med 1993 5 977 868366922

